# Anatomy, Descriptive and Surgical

**Published:** 1878-10

**Authors:** 


ANATOMY, DESCRIPTIVE AND SURGICAL. By Henry Gray, F.R.S., Fellow of the Royal College of Surgeons, and Lecturer on Anatomy at St. George's Hospital Medical School. With five hundred and twenty-two engravings on wood. The drawings by H. V. Carter, M.D., and Dr. Westmacott. The dissections jointly by the author and Dr. Carter. With an Introduction on General Anatomy and Development. By T. Holmes, M. A. Cantab, Surgeon to St. George's Hospital. A new American, from the eighth and enlarged English, edition. To which is added, Landmarks, Medical and Surgical. By Luther Holden, F.R.C.S., Surgeon to St. Bartholomew's and the Foundling Hospital. Philadelphia: Henry C. Lea. 1878.

Gray's Anatomy has become a leading text-book in Medical Colleges, and this new American edition will doubtless be sought by learners.

